# Surface Activity and Efficiency of Cat-Anionic Surfactant Mixtures

**DOI:** 10.3389/fchem.2021.790873

**Published:** 2021-12-14

**Authors:** Camillo La Mesa, Gianfranco Risuleo

**Affiliations:** ^1^ Department of Chemistry, Sapienza University of Rome, Rome, Italy; ^2^ Department of Biology and Biotechnologies Charles Darwin, Sapienza University of Rome, Rome, Italy

**Keywords:** cat-anionic surfactants in mixture, surface adsorption, micelle formation, bulk interaction parameters, surface interaction parameters, regular solution theory

## Abstract

The surface activity of surfactant mixtures is critically analyzed. Cat-anionic systems, in which two ionic species are mixed in non-stoichiometric ratios, are considered. With respect to the solution behavior, where a substantial decrease of *cmc* is met compared to the pure components, a moderate effect on surface tension, γ, occurs. Compared to the pure species, the decrease of surface tension for such mixtures is not significant, and no clear dependence on the mole fraction anionic/cationic is met. The surface tension is grossly constant in the whole concentration range. Conversely, the interaction parameter for surfaces, *β*_*surf*_ (calculated by the regular solution theory), is more negative than that for micelle formation, *β*_*mic*_. This fact suggests that the desolvation of polar heads of the two species at interfaces is largely different. Very presumably, the underlying rationale finds origin in the sizes and solvation of both polar head groups.

## 1 Introduction

Washing and cleaning activities in the B.C. ages are reported in Egyptian, Greek, and Roman manuscripts, but the use of surfactants in those times were not only limited to the aforementioned purposes. Over 5,000 years ago, in the era of the first Egyptian kingdom, calcium-soaps in paste form were used as a lubricant for axles of carts, and their source remains unknown (Whitaker, 1965). Later on, ancient Greeks used potash, or soda lime, to clean fabrics, but various oils for cleaning their skin. One such example is the encounter of Ulysses with Nausicaa in the sixth chapter of the *Odyssey*, Wherein young Nausicaa is washing linen and togas in the sea around her native island. The beautiful description of that meeting is a clear-cut indication that the Greeks did not use soap for fabric cleaning, very presumably because of the strong salinity inherent to sea waters. The ancient Greeks were not fully aware of the technicalities required to produce hard-water compatible soaps from edible oils. In more recent times, the Franks hydrolyzed beef fat with KOH (or, more reasonably, with K_2_CO_3_), to get what is known as “*Marseille*” soap, also containing some glycerol. As reported in “*De Bello Gallico*”, the Franks shocked the contemporary Romans by the extensive use of soap in hair cleaning (Caesar and Winterbottom, 1983).

Let us leave the history of surfactants, and notice that the surfactants actually in use today only became ubiquitous in recent times, when fatty acid soaps, as oleates, palmitates, and laurates, were replaced by synthetic species. The first synthesis of alkyl sulfates and sulfonates dates to the 1930s (Barkenbus and Owen, 1934; Caesar, 1983). It was later followed by that of alkyl poly-oxyethylene glycol mono-ethers (Schick, 1963), and by species of the like. The same holds for the synthesis of surfactants from natural sources (Mäki‐Arvela et al., 2007; Kraus and Lee, 2012; Chowdhury et al., 2020).

**FIGURE 1:**
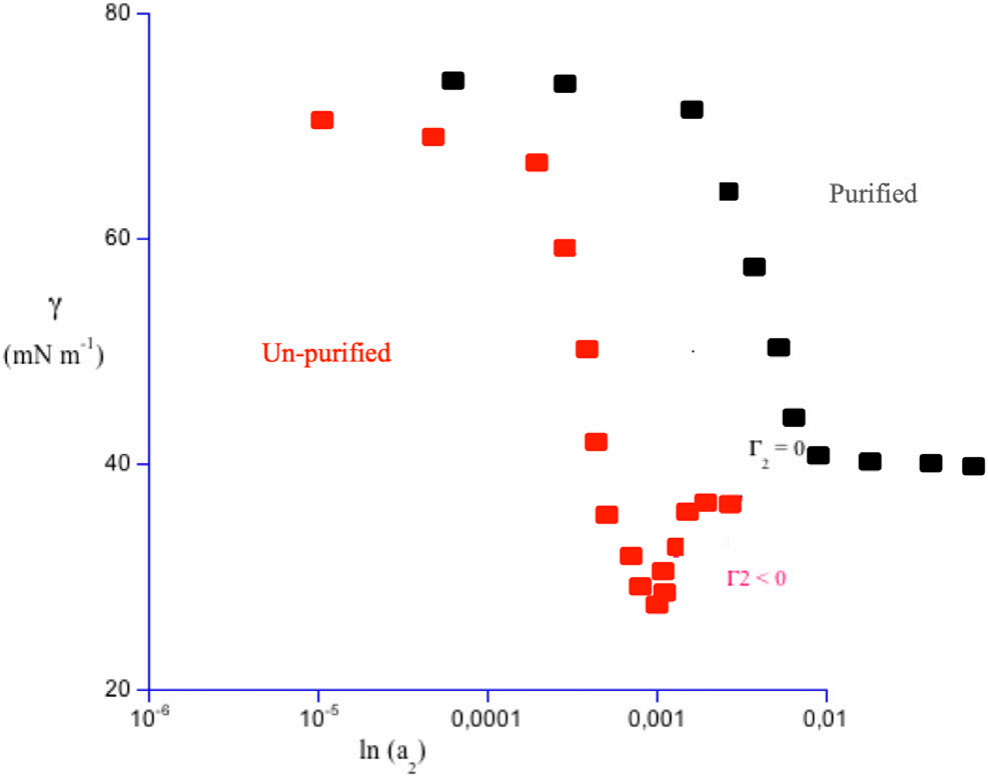
Plot of surface tension, γ, in mN m^−1^, versus solute activity, a_2_, in logarithmic scale and in arbitrary units, for a commercial sodium aryl-alkyl sulfate containing 1.0 w% octanol, in red. Γ_2_, the tangent of γ *vs.* ln a_2_, is practically null only well above the critical micellar concentration, *cmc*. Around that threshold, it can be null, positive, or negative. Surface activity in un-purified systems spans a concentration range and differs from that of the pure surfactant, black symbols. The low concentration regime is followed by a saturation one. There γ reaches a real plateau when a_2_ is higher than the *cmc*. In purified samples, thus, both the minimum and the inversion in the sign of Γ_2_ do not occur. Above the *cmc* the interface is surfactant-saturated and micelles begin to form.

Characterization of surface activity was systematically performed by the Du Noüy ring (Lunkenheimer et al., 1981), or pendant drop (Stauffer, 1965) methods. Despite the simplicity of such methods, based on classical mechanics and still in use, characterization was reliable. Experiments on raw surfactants indicated the occurrence of a pronounced surface tension minimum at concentrations close to the *cmc*, the critical micellar concentration. In fact, species more surface-active than surfactants, such as fatty acids, or long-chain alkanols, are present as impurities (McBain and Davies, 1927). In non purified species, a decreasing surface tension regime is observed at low concentrations, with the presence of a pronounced minimum at the critical micellar concentration. That behavior is followed by a constant surface tension regime, Figure 1.

Such behavior is not consistent with the thermodynamics of fluid surfaces, based on the Gibbs adsorption isotherm. Thanks to the perseverance of K.J. Mysels, the effect was explained (Elworthy and Mysels, 1966).

Mysels continued work on bubbling and de-foaming surfactant solutions, whose foams are rich in more surface-active species (fatty acids, or long-chain alkanols) until the surface tension minimum vanished (Mysels, 1986). Note that a decrease in surface tension is also obtained in solutions of alkali metal carboxylates at concentrations close to the *cmc* by bubbling CO_2_, and favoring the basic hydrolysis of carboxylates. One obtains a colloidal buffer of fatty acid and its salt. This is why high-quality, acid-depleted, soaps are obtained by titration of fatty acids in absolute ethanol with strong bases, such as K, or Na, ethoxide (Glass, 1971). Remember, too, that calcium ions favor the precipitation of alkyl carboxylates. This is at the basis of the so-called “hydrometry”, a method used to determine “*water hardness*” (Pereira et al., 2012).

In what follows we report on surfactant mixtures of oppositely charged species. Expectedly, these systems should give a promising surface activity behavior, much more substantial than those actually in use. For these reasons, we focused first on systems made by one surfactant only. Later, we focused on cat-anionic systems and proceeded to link the significant changes that are observed in bulk (with the formation of micelles at very low concentrations) with those that do presumably occur at the air-water interface.

## 2 Surfactant Performances

Formulations presently in use try to get the best surfactant performances. However, what is meant by “*best surfactant performances*” is elusive. To reduce the amount of surfactants in molecular/ionic form in the bulk, we rely on the “*hydrophobic effect*”, expressing the difficulty of dissolving hydrocarbon moieties in water (Tanford, 1980). The onset of a *cmc* is controlled by the above effect. Therefore, the constancy of surface activity is preliminary to surfactant aggregation.

Due to the dual nature inherent to surfactants [Hartley defined them *schizophrenic* molecules (Murray and Hartley, 1935)], they tend to avoid water and favorably partition on the surface, as evidenced by an excess concentration therein. When surfaces saturation is no longer possible, the above species aggregate in the bulk and form micelles, to minimize contact with water. Micelle formation ensures that:

1) The constancy of surface tension is attained;
2) The concentration of surfactant in molecular form is kept constant;
3) The interactions of surfactant species with water are minimized.

These are not their only peculiar features.

If we do not consider balanced micro-emulsions (Ruckenstein, 1978; Pitt et al., 1996; Ruckenstein, 1996), the minimum surface tension of such formulations in aqueous media, γ, is never ≤20 mN m^−1^. Micro-emulsions, however, are not convenient for practical purposes. The mentioned γ threshold is never attained if only one surfactant is used, irrespective of additives, as adding co-surfactants, or salts.

To overcome such unescapable drawbacks, formulations based on surfactant mixtures have been proposed. The most promising are defined as *cat-anionics* (Khan and Marques, 1997; Kronberg, 1997) since they contain both anionic and cationic species. We do not consider here mixtures of ionic and nonionic species, reported in selected articles (Jańczuk et al., 1995; Mulqueen and Blankschtein, 1999). *Cat-anionic* species do not significantly decrease the surface tension. The mentioned threshold is a sort of “*Hercules*’ *pillar*”, and it is not possible to go beyond it. This holds in aqueous solutions, even though it is possible to achieve very low γ values in micro-emulsions and at water-oil interfaces. The reason for this is inherent to the very nature of surfactants, saturating air-water, or water-oil, interfaces (Chanda and Bandyopadhyay, 2006; Müller et al., 2021), but still retaining a finite area. Such behavior is controlled by solubility, packing at interfaces, film elasticity, the orientation of polar part(s) toward the bulk, etc. [*N.B.* Surfactants at interfaces orient as polarity-sensitive chemical dipoles, which they are]. Some such points shall be described in the following sections.

## 3 Some Preliminary Aspects

We report below the fundamentals of surfactants, try to reduce the surface tension of water-based systems, and try to predict what one could achieve using surfactant mixtures. Let us consider first systems made of water (or brine) and a single surfactant. The following relation links the bulk to the surface activity.

$$\mathrm{dG=}\sum_{\mathrm{i=1}}\mu_{i}dn_{i}+\mathrm{\gamma dA-SdT+}V_{a}\mathrm{dP+}V_{b}\mathrm{dP}$$ (1)

At T and P cost, dG reduces to

$$\mathrm{dG=}\sum_{\mathrm{i=1}}\mu_{i}dn_{i}+\mathrm{\gamma d}A$$ (1')

At equilibrium, the above equation, termed Gibbs adsorption isotherm, results to be

$$0=\sum_{i=1}n_{i}d\mu_{i}+Ad\gamma$$ (2)

For a system made of solvent and only one surfactant, Eq. 2 reduces to

$$\left(n_{2}/A\right)=\Gamma_{2}=-\left(\mathrm{d\gamma /d}\mu_{2}\right)$$ (3)

There *A* is the area onto which the surfactant spreads (in nm), and Γ_2_ (moles area^−1^) is the surface excess concentration of the solute with respect to the bulk. That of the solvent, Γ_1_, is set equal to 0. Γ_2_, an inverse partial molal quantity, is proportional to (dγ/dμ_2_). Eq. 3 indicates how the surface tension efficiency changes with composition and vanishes above the *cmc*. Implicit in Eq. 3 is the fact that γ is not 0, whereas (dγ/dμ_2_) can be such. This fact implies that the solute activity, a_2_, is constant above the *cmc*. The above considerations lead many to define the so-termed “(pseudo) *phase separation approach*” to micelle formation (Shinoda and Hutchinson, 1962; Moroi et al., 1984; van Os et al., 1991). In that approach, the *cmc* is considered in analogy with the onset of a “*micellar* (*pseudo*)*phase*”. Micelle size, therefore, is immaterial in this approach. For simplicity, we do not consider here approaches based on the mass action approach, or more refined ones (Corkill et al., 1969; Kamrath and Franses, 1984).

Note that the relation linking the energy gain inherent to micelle formations (dG_mic_) is a solubility product, and, neglecting charge effects, dG_mic_ = RT ln*cmc.*

**FIGURE 2:**
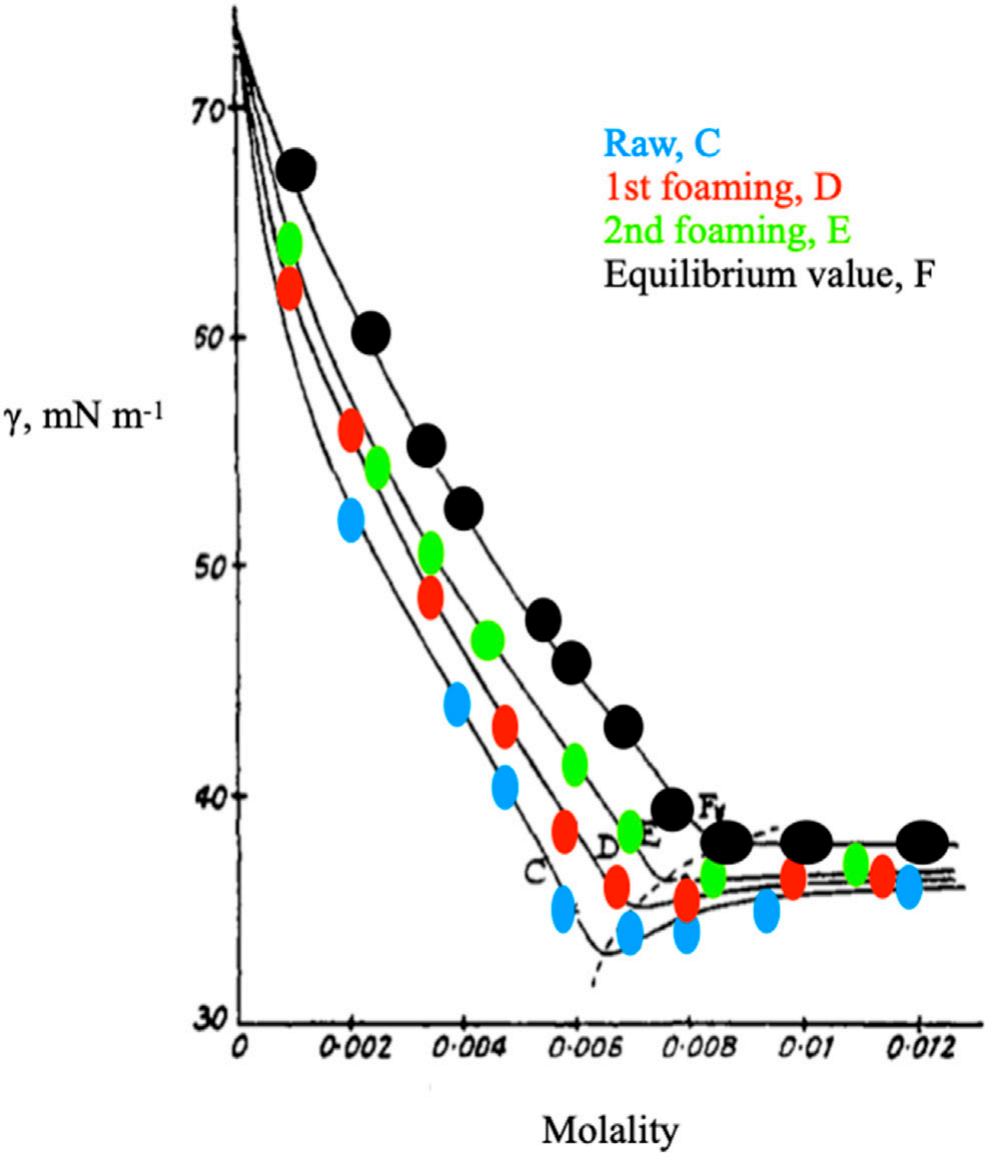
Data redrawn from Ref.s (Elworthy and Mysels, 1966; Mysels, 1986), showing surface tension changes by foam purification. The dotted line indicates the shift of the *cmc* upon progressive de-foaming. The *cmc* value inferred by ionic conductance is 8.21 10^−3^ mol kg^−1^, at 25.00°C. It corresponds to the inflection point of the curve indicated by black points and is obtained with highly purified sodium dodecylsulfate, SDS.

**FIGURE 3:**
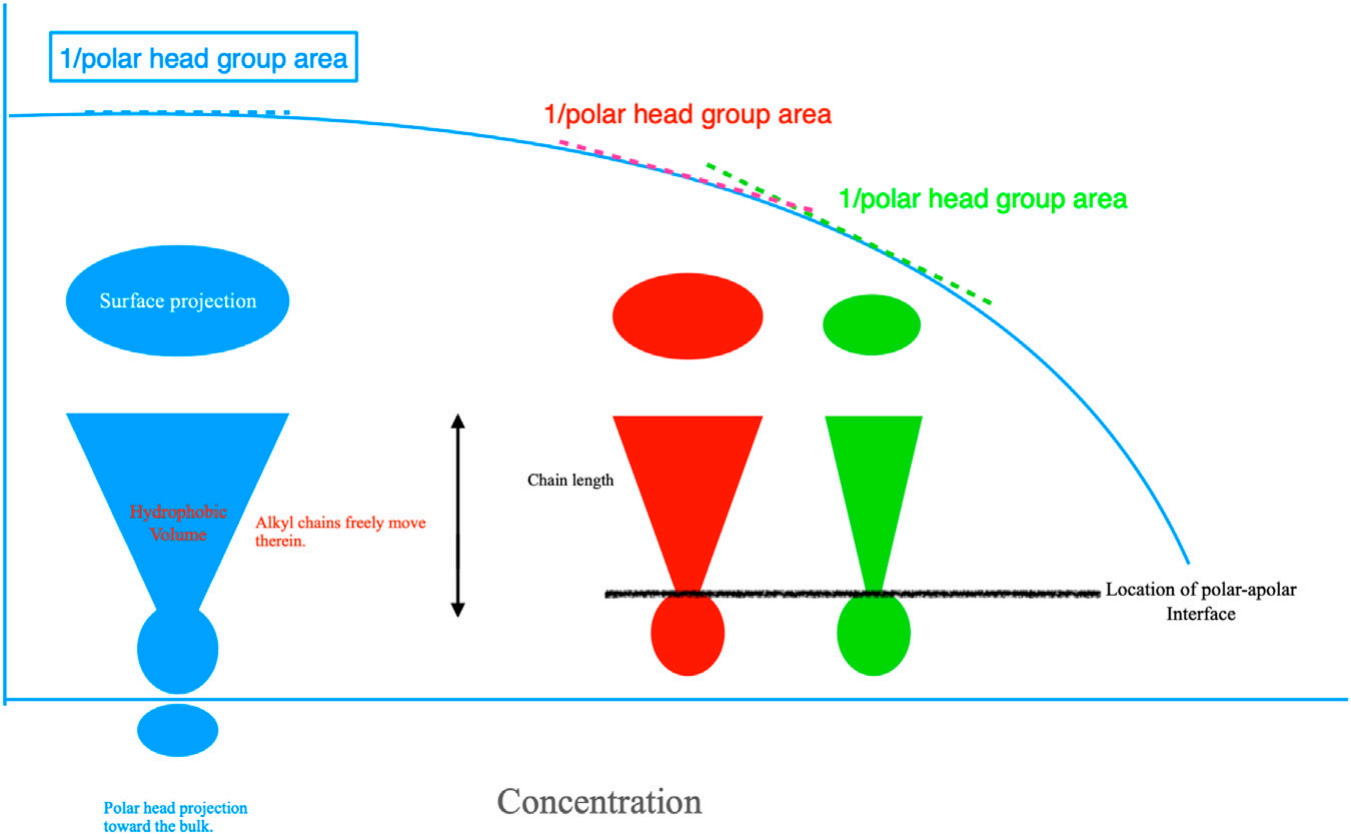
Left; cut-view projection of a surfactant at the air-water interface. Chains are hidden into cones. The polar head projection toward the bulk decreases as a direct consequence of composition. The same holds for the surface projection, whose value is inversely proportional to Γ_2_. The higher is it, the lower the surface area. Surfactants pack more or less densely in mono-layers of width equal to the alkyl chain length and pass from a liquid expanded to a liquid compressed state until a compact layer is attained. That behavior corresponds to a liquid-solid phase transition. Red and green cones overlapping with the γ *vs.* concentration plot, in light blue, contain an alkyl chain of the same length as the blue one, but in more compact form; this implies that the surface projection is lower. Alkyl chains are like whips, with polar areas anchoring them at the polar-apolar interface. The degrees of motion of the alkyl chains do reduce in direct proportion to the cone(s) volume.

The links between quantities reported in Eqs. 2, 3 are drawn in Figures 2, 3. The surface tension may decrease slightly above the *cmc* (La Mesa and Ranieri, 1993), but never reaches zero. For practical purposes, we assume that the surface tension is constant above the *cmc*. The real problem to face is to minimize γ, or maximize the surface pressure, π (γ° - γ, > 0, γ°) being that of the solvent.

The addition of electrolytes increases surface adsorption. In fact, water becomes less capable of dissolving the surfactants. The interface area is progressively reduced; this favors an efficient packing of alkyl chains in monolayers, still keeping the chain length constant. Surfactants orient at interfaces and behave as surface-anchored whips, with non-polar regions facing toward the air. Their rotational degrees of freedom drastically reduce when surfaces are saturated, Figure 3. These statements do not imply the disappearance of γ.

A reduction in the number of charges at interfaces modifies the surface charge density, the molecular packing, and double-layer effects, as well. Direct consequences are manyfold, and not only positive. A decrease in surface charge density implies a decrease in surface area and grants the occurrence of a stiff monolayer (not far from being close to a compressed state), and a quite low film elasticity.

This implies that foam stability is controlled by film elasticity and disjoining pressure; the contributions due to each term depend on film type, surfactant nature, and co-solute concentration (Hédreul and Frens, 2001; Georgieva et al., 2009). Provided Eq. 3 contains a chemical potential for each component, the relations for ternary, or multicomponent, surfactant systems have exactly the same functional form as the previous ones.

**FIGURE 4:**
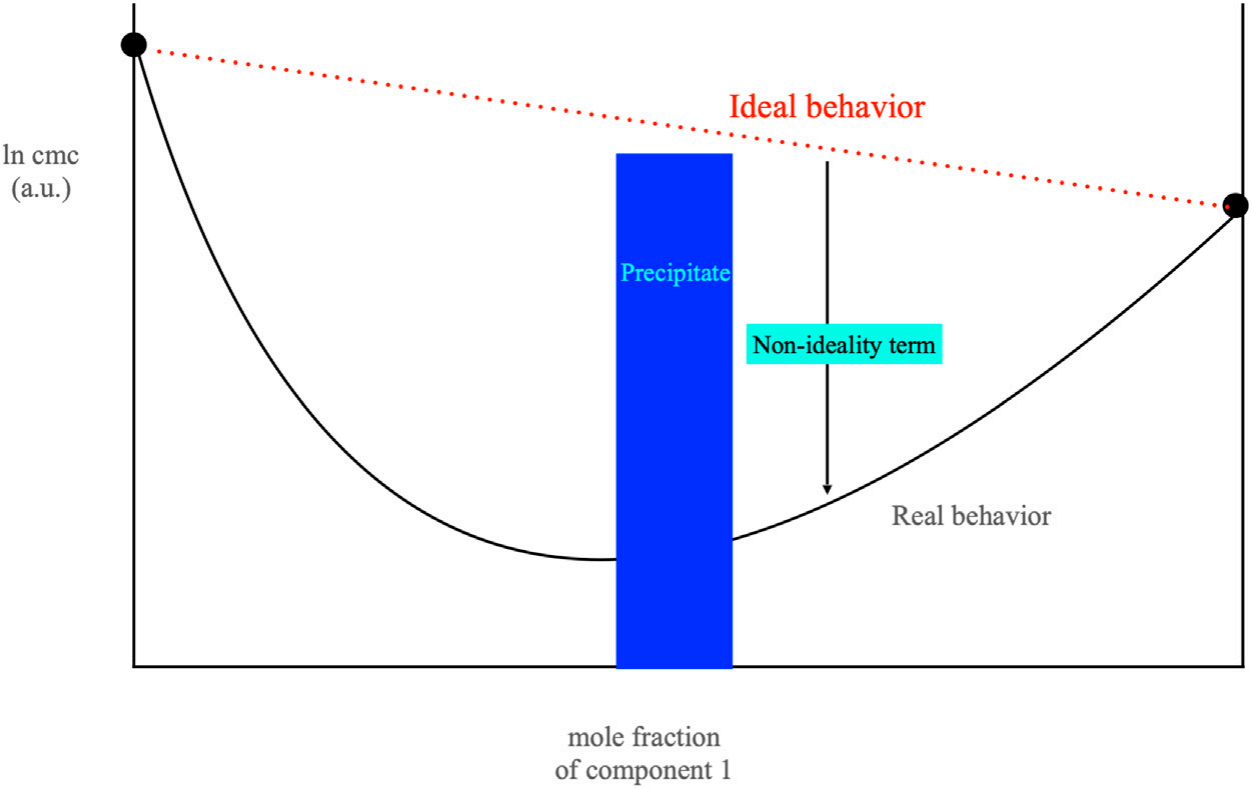
Plot of the *cmc of* a cat-anionic mixture, in arbitrary units, *vs.* the mole fraction of the first surfactant component. The blue rectangle in the center of the figure indicates the location of the precipitate area, where *Cat* ^*+*^ *An* ^*−*^ solids occur. The red dotted line indicates the behavior expected if ideal mixing were to occur whereas the down-pointing arrow (in black) indicates the non-ideal effects. Data are based on the sodium dodecylsulfate/cetyltrimethylammonium bromide system, at 25.0°C. Component 1 in the plot is sodium dodecylsulfate. The black line indicated as Real behavior was drawn imposing the *ß* parameter (of value −21.0 kJ mol^−1^) to be constant.

The formation of cat-anionic micelles assumes the non-ideality of mixing among two oppositely charged surfactants. Micelle formation and *cmc*’s do not regularly match with composition, large departures from the “*ideal*” behavior are met (Moroi, 1992; Raghavan et al., 2002). A pronounced minimum in *cmc* values vs. mole fraction occurs as per Figure 4. Unexpectedly, this does not imply that the surface tension behaves accordingly. γ values above the *cmc* remain grossly constant in a wide mole fraction range, centered around the 1/1 mole ratio*.* The behavior of bulk and surface phases is controlled by the surfactant (s) partition. Matter transfer from one phase to another is interrelated since micelle onset is subsequent to surface saturation. The relations between bulk and surface phases are not understood if the interface area occupied by surfactants is neglected.

The molecular area of long-chain alkanols is less than 0.2 nm^2^ (Lo Nostro and Gabrielli, 1993), that of most surfactants is, at least, twice larger (Li et al., 1999; Hore et al., 2005; Menger et al., 2009). The surfactant packing at interfaces is limited by their molecular features. Swamping electrolytes reduce the polar areas but do not cancel them. The intrinsic sizes of chains attached to the polar group rule out such an eventuality [N.B. The term “intrinsic sizes” would be better replaced with the surfactant(s) projections at interfaces].

An important point must be considered. The stability of surfactants into micelles is controlled by electrostatic (Nagarajan, 1986), hydrophobic contributions (Maibaum et al., 2004), curvature elasticity of the bi-layers [in case of vesicles (Safran et al., 1990; Jung et al., 2002)], and other terms, granting a significant growth in micelle size, with the formation of cylinders, or other supramolecular structures (Mukerjee, 1980; Dill and Flory, 1981; Ninham and Evans, 1986). Such shape transitions are governed by the “*packing constraint*” (Israelachvili et al., 1976).

The behavior at interfaces is different. To put evidence behind these statements: the molecular area of dodecanol at the air-water interface is close to 0.2 nm^2^ (Vollhardt et al., 2000), SDS in the range 0.43–0.46 (Purcell et al., 1995; Tah et al., 2011), that of alkyl poly-oxyethylene glycols from 0.38 to 1.20 [depending on alkyl and PEO chain length (van Os et al., 1993)], that of CTAB between 0.52 and 0.72 nm^2^ (Biswas et al., 2006; Yazhgur et al., 2018), and so on. Considering some reduction in case of partial, or total, charge neutralization of the surfactant ions, it is un-conceivable to get much lower molecular areas, but it is possible to get dense packing and formation of “solid” monolayers.

## 4 Cat-Anionic Mixtures: Some Thermodynamics

The molecular schizophrenia of ionic surfactants drastically increases when they are mixed with an oppositely charged analog. Surface areas are slightly modulated by the co-presence of the two species, but the bulk association is largely modified with respect to the corresponding binary systems (Jónsson et al., 1991; Wang et al., 2007; La Mesa and Ranieri, 2019; Peyre, 2009; Long and Hao, 2012). A drastic decrease of *cmc* values, measuring some orders of magnitude, is observed, as shown by Figure 4. It is also common the occurrence of a precipitate when the [anionic/cationic] charge ratio, *R*, is close to unity. The latter behavior is ascribed to the metathesis of mobile ions with long-chain ones, and the formation of a hydrophobic ionic solid. The latter is thermotropic (thermo-sensitive) in character rather than lyotropic (water-soluble) (Khan and Marques, 1999).

As to the solution behavior, we assume micelle formation to be a phase separation. For two and three species, respectively, the *cmc* in a mixed system is defined as (Holland et al., 1991; Muzzalupo et al., 2006)

$$cmc_{\mathrm{mix}}=\left[\left(\gamma_{2}cmc_{2}\right)\left(\gamma_{3}cmc_{3}\right)\right]/\left[\left(\gamma_{2}X_{2}cmc_{2}\right)\left(\gamma_{3}X_{3}cmc_{3}\right)\right]$$ (4)

There, *cmc*_mix_ is the mixture critical value in the given conditions, *cmc*_2_ and *cmc*_3_ are the concentrations of the pure species, γ_2_ and γ_3_ the activity coefficients of the related surfactants; *X*_i_’s are the mole fractions. The equation is the multiplier in γ_i_ *cmc*_i_’s over the summation of the same variables. In the phase separation approach, dG is calculated by (Eq. 4). Its a-dimensional form, (∆G_mix,mic_/RT) = *cmc*_mix_, simplifies the calculations, mostly if the inverse of Eq. 4 is considered.

Eq. 4 reduces to ln [(γ_2_ *cmc*_*2*_) (γ_3_ *cmc*_3_)] − ln [(γ_2_X_2_ *cmc*_2_) + (γ_3_X_3_ *cmc*_3_)]; the latter is a reference value for micelle formation. Eq. 4 accounts for the non-ideality of mixing both for the molecular and micellar forms. Experimental *cmc*’s allow for acquiring the excess Gibbs energy of micelle formation, (∆G_mix,mic,exc_/RT) = ln (γ_2_γ_3_) − ln(γ_2_ + γ_3_). Precise relations rely on the “*regular solution theory*” (Hildebrand et al., 1970), accounting for non-ideality effects. After some straightforward algebra, one gets the relation for the activity coefficient of the *i*th solute, γ_i_, expressed as

$$\gamma_{i}=\exp-(\beta {X_{i}}^{2})$$ (5)

where β is the so-called “interaction parameter”. More developments can be introduced if β does not depend, or slightly depends on the composition. In the former eventuality

$$\beta_{\mathrm{mic}}=\Delta G_{\mathrm{mix,mic,exc}}\left[{X_{2}}^{2}+{X_{3}}^{2}/{X_{2}}^{2}{X_{3}}^{2}\right]$$ (6)

The above statements are on the basis of all energy calculations. The (β/∆G_mix,mic,exc_) ratio that is obtained scales according to the regular solution approach, see Eq. 6. The latter is exhaustively discussed by Rubingh (Rubingh, Mittal). In addition, β < −1, and decreases in direct proportion to the system non-ideality. No exceptions to this statement are reported. In a mixture of sodium decylsulfate, SDeS, and decyltrimethylammonium bromide, DeTAB, for instance, β = −18.5 RT units (Holland and Rubingh, 1983; Lozano et al., 2011). Since chain lengths are the same, it is conceivable that ionic interactions among polar head groups play a dominant role in such processes, Figure 4.

## 5 Interface Properties

Cat-anionic solids spread at interfaces (Tah et al., 2012; Barbetta et al., 2014), as also do their non-stoichiometric mixtures. Such mixtures give a quite low permeability to air (Jurašin et al., 2017; Olechowska et al., 2019) and find application in the area of foams. The synergism in mixed systems also implies a large reduction in both surface tension efficiency and effectiveness. This is not exactly true. The calculated parameters, β_surf_ and β_mic_, respectively, are somehow related to the interaction’s modes between surfactants in mixed micelles and in mixed monolayers, too. Investigation can determine whether these systems are synergistic in some aspects. Values required for clarifying the effectiveness of such theories are:

1) The surface tension *vs.* log *c* plots of the individual species close to their *cmc*’s;
2) The critical micellar concentration of at least one surfactant mixture.

**FIGURE 5:**
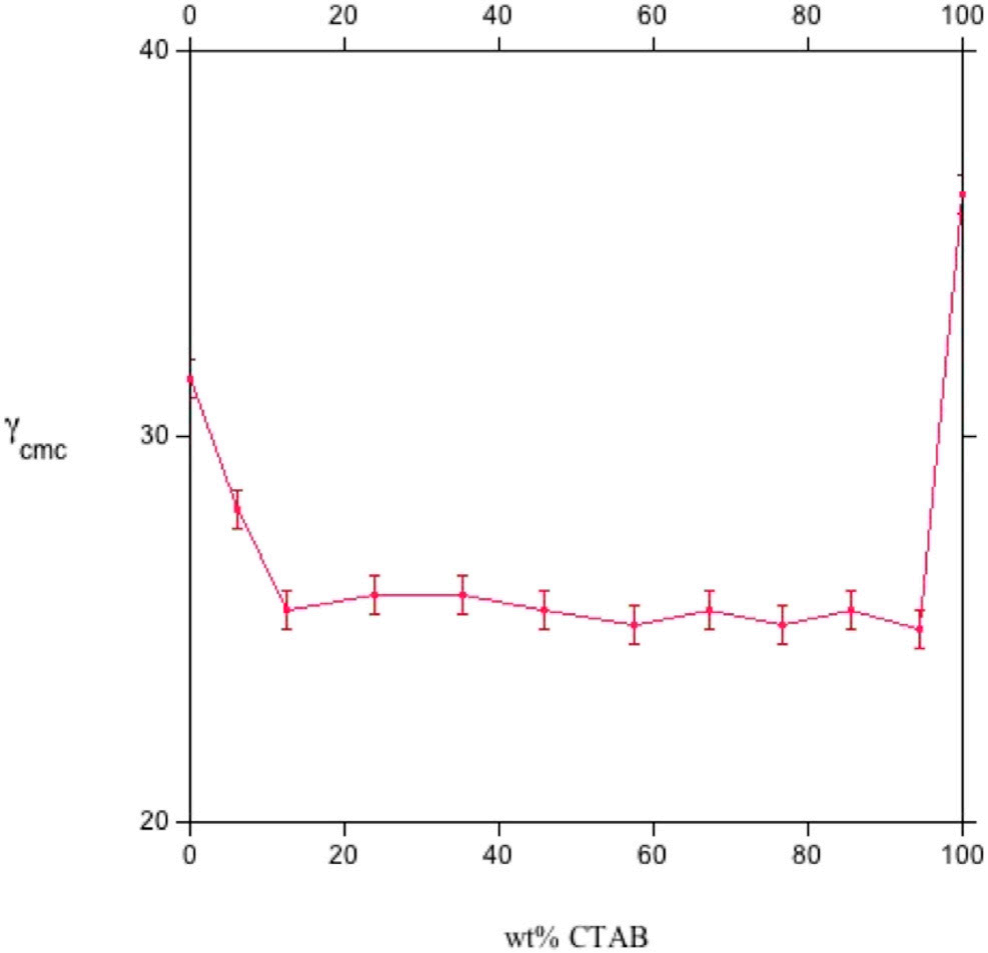
The surface tension at the *cmc*, γ_cmc_ (in mN m^−1^), *vs.* CTAB wt% for sodium octylsulfate/cetyltrimethylammonium bromide mixtures, at 25.0°C. The central part of the plot is characterized by nearly constant surface tension, oscillating around 25 mN m^−1^. The plot was redrawn by data in Ref. (Comelles et al., 2015).

**FIGURE 6:**
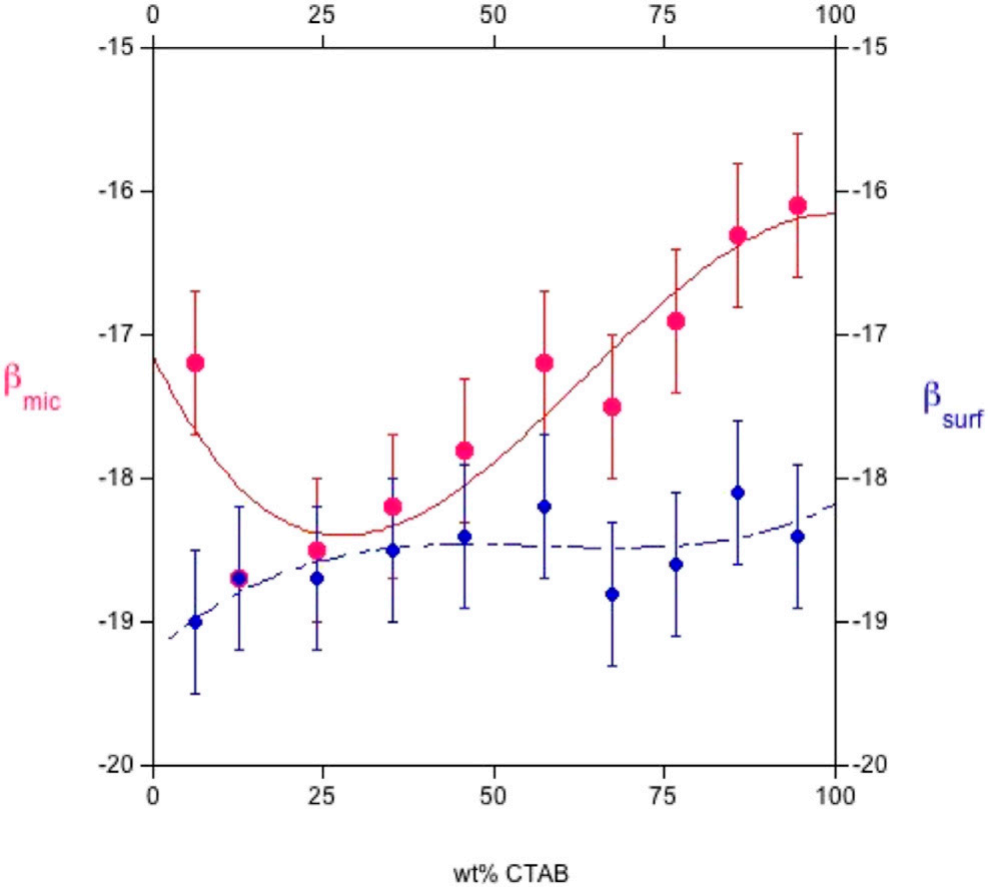
Plot of the interaction parameters, β_surf,_ and _mic_, respectively, at the *cmc*, *vs.* the CTAB wt% in the system sodium octylsulfate/cetyltrimethylammonium bromide at 25.0°C. *ß* parameters are in kJ mol^−1^. β_surf_, in blue, is always more negative than β_mic_. The plot was drawn by elaborating the data given in Ref. (Comelles et al., 2015).

The concentration of a mixture producing a surface tension close to that attained by individual surfactants must also be known. In this way, the chemical structure and molecular environment of the f β_surf_ and β_mic_ values are quantified. Pertinent data are reported in Figures 5, 6.

Rosen et al. related the regular solution theory to bulk and surface phases. The relations they developed are expressed as

$$\beta_{\mathrm{mic}}=\mathrm{In}\left(a_{1}C_{\mathrm{12mic}}/X_{\mathrm{1mic}}C_{\mathrm{1mic}}\right)/{\left(\mathrm{1-}X_{\mathrm{1mic}}\right)}^{2}$$ (7)

$$\beta_{\mathrm{surf}}=\mathrm{In}\left(a_{1}C_{12}/X_{1}C_{1}^{0}\right)/{\left(1-X_{1}\right)}^{2}$$ (8)

where a_1_ is the mole fraction of species one in the given medium, the same holds for C_1_ and X_1,mic_. C_12_ refers to the mole fraction of the 1–2 mixture. The ratio among two such quantities correlates to the concentrations of the different physical states, be they bulk or surface ones.

Such results indicate that β_surf_ is more negative than β_mic_, although both are calculated by the same theory (*N.B.* The regular solution theory, in fact, is model-dependent). The occurrence of strong synergism in monolayer films occurs, and the non-ideality effects are higher than in aggregates. Presumably, the interactions between surfactants are more favorable at the air/aqueous solution interface. Note, too, that the absolute values of β_surf_ and β_mic_ depend on temperature. They are larger in the cationic-rich than in the anionic-rich side, presumably because of the polar head-groups hydration. In fact, SDS is more hydrated than CTAB. As a result, electrostatic attraction in anionic-rich mixtures is more significant than in cationic-rich ones, mostly when T is raised. This result suggests a major effect on ions at the air/water interface rather than in the bulk. We are aware that such a simplified approach is questionable in a complete rationalization of the observed behavior.

The surface tension of selected non-ideal cat-anionic mixtures was investigated (Rosen and Hua, 1982; Szymczyk, 2013; Pinazo et al., 2020). Rosen et al. (Liu and Rosen, 1996; Rosen et al., 2005; Zhou, Rosen; Rosen and Kuniappu, 2012) developed a theory predicting a significant surface tension reduction and got innovative results. Among the systems that are reported we consider triethanolammonium dodecyl-dioxyethylene sulfate, TADPS, with dodecyltrimethylammonium bromide, DTAB, CTABr, or hexadecylpyridinium chloride, CPCl, respectively. It is not our intention to discuss the equations, the synergism in surface tension effectiveness, or to show the relations between the expressions derived by Rosen et al. The surface tension at the *cmc* is obtained for a given species system under special conditions and is defined as “*surface tension reduction effectiveness*”. But, although low *cmc*’s are reached for matched systems, no significant reduction in surface tension occurs.

The problem one has to face relies on the fact that β_surf_ and β_mic_ are strictly interrelated. If we consider that the reference β value usually refers to the bulk state, and is based on the regular solution theory, it results that β_surf_ is strictly related to β_mic_, and suffers from the same drawbacks. Accordingly, the pertinent equations are strongly model-dependent.

All surface properties are related to those in the bulk, whose activity coefficients fulfill the regular solution theory. Values relative to the CTAB-SOS system (Comelles et al., 2015) indicate that β_surf_ and β_mic_ oscillate in the range between −16 and −19 kJ mol^−1^, as reported in Figures 5, 6. It results that β_surf_ is more negative than β_mic_, i.e., adsorption is more energetic than the bulk behavior. Perhaps, although the formation of mixed micelles is significantly affected by the mole fraction, the same cannot be said for surface tension.

In terms of “*surface effectiveness*”, therefore, the use of cat-anionic mixtures is partially ineffective. Perhaps *Cat-An* mono-layers are much more efficient than those found in other systems. To bring to mind but a few, note that film elasticity and disjoining pressure of foams based on *Cat-An* mixtures are more effective compared to single-component surfactant systems. The reported γ values are comparable to those of fully fluorinated surfactants, which show exceptionally low surface tension compared to all hydrocarbon-based species (La Mesa and Sesta, 1987).

## 6 Conclusion

Originally, the interest toward such systems arose from attempts to model mono-layers and lamellar phases of modulated surface charge density (Jónsson et al., 1991; Khan and Marques, 1999). Data reported so far deal with mixed micelle formation, on the related thermodynamic features, on the phase separation of 1–1 systems, on the formation of cat-anionic solids, on vesicles onset, and on their interactions with polymers/biopolymers, as well (Mal et al., 2018; Barbetta et al., 2011; Bonincontro, Falivene, La Mesa, Risuleo, Ruiz Peña).

As to surface activity, apart from data presented by Rosen (Rosen and Hua, 1982; Liu and Rosen, 1996; Rosen et al., 2005; Zhou, Rosen; Rosen and Kuniappu, 2012) dating from the 80s’, the number of contributions to such fields are poor (Lozano et al., 2011; Comelles et al., 2015; Yazhgur et al., 2018), the paucity of studies in the reference section of this very paper are proof of that. This is astonishing since many contributions were issued on their association features. Conversely, surface tension data were considered as ancillary results. For mixtures made of hydro- and fluorocarbons, for instance, no surface tension data are available. Thus, we do not know the relevance of cat-anionic films in foam preparation, although this aspect is relevant for many practical applications.

There are several major points in the use of cat-anionic systems as surface-active compounds which deserve consideration. Some are obvious, others are still a matter of controversy. As to their use in the optimization of surface properties, a lot can be said. In particular, β_surf_, the surface interaction parameter, is more negative than the one in the bulk. Unexpectedly, the effect that one observes in the formation of mixed micelles is by far more substantial than at surfaces. This fact is rather counterintuitive if we consider that the effectiveness in surface tension reduction is nearly constant in the mole fraction range which has been investigated. Almost nothing is known on the surface tension of cat-anionic mixtures containing hydrocarbon and fluorocarbon surfactants. Thus, efforts to go beyond are somehow not actionable. It is left to future work to proceed along this line.
